# Silencing of MsD14 Resulted in Enhanced Forage Biomass through Increasing Shoot Branching in Alfalfa (*Medicago sativa* L.)

**DOI:** 10.3390/plants11070939

**Published:** 2022-03-30

**Authors:** Lin Ma, Yongchao Zhang, Hongyu Wen, Wenhui Liu, Yu Zhou, Xuemin Wang

**Affiliations:** 1 Institute of Animal Sciences, Chinese Academy of Agricultural Sciences, Beijing 100193, China; malin@caas.cn (L.M.); wenhognyu@163.com (H.W.); 2 Key Laboratory of Superior Forage Germplasm in the Qinghai-Tibetan Plateau, Qinghai Academy of Animal Science and Veterinary Medicine, Qinghai University, Xining 810016, China; zhangych06@126.com (Y.Z.); quhliuwenhui@163.com (W.L.); 3 Institute of Characteristic Crops Research, Chongqing Academy of Agricultural Sciences, Chongqing 402160, China; xinganmermer@163.com

**Keywords:** alfalfa, MsD14, strigolactone, shoot branching, forage biomass

## Abstract

Branching is one of the key determinants of plant architecture that dramatically affects crop yield. As alfalfa is the most important forage crop, understanding the genetic basis of branching in this plant can facilitate breeding for a high biomass yield. In this study, we characterized the strigolactone receptor gene *MsD14* in alfalfa and demonstrated that *MsD14* was predominantly expressed in flowers, roots, and seedpods. Furthermore, we found that *MsD14* expression could significantly respond to strigolactone in alfalfa seedlings, and its protein was located in the nucleus, cytoplasm, and cytomembrane. Most importantly, transformation assays demonstrated that silencing of *MsD14* in alfalfa resulted in increased shoot branching and forage biomass. Significantly, MsD14 could physically interact with AtMAX2 and MsMAX2 in the presence of strigolactone, suggesting a similarity between MsD14 and AtD14. Together, our results revealed the conserved D14-MAX2 module in alfalfa branching regulation and provided candidate genes for alfalfa high-yield molecular breeding.

## 1. Introduction

As one of the most important forage crops, alfalfa, with a global hay market of 8.3 million metric tons in 2017, plays a critical role in dairy and meat production [1]. The high biomass is a key consideration for the market acceptance of a newly developed alfalfa cultivar [2]. Therefore, extensive studies are urgently needed to improve the biomass yield in the alfalfa industry. As the genetic and environmental factors affecting branching impact biomass yield significantly, the identification and characterization of branching-related genes are indispensable for understanding the genetic basis of branching and facilitating high biomass yield breeding in alfalfa [3].

Branching is one of the key determinants of plant architecture and affects important plant functions, such as light interception and resource use [4,5]. Plant branches control bud initiation and outgrowth, which are regulated by a global and complex regulatory network of genetic, hormonal, and environmental factors [6,7]. Among these factors, hormones, including auxins-, cytokinins-, and brassinosteroids, have been intensively illustrated to regulate branching [8,9,10]. In general, auxin is synthesized in the shoot apex and transported basipetally to inhibit the activation of axillary buds and hence shoot branching, while cytokinins represent a possible second messenger for auxin signaling that activates buds by regulating the transcription of relevant genes in the bud [11,12,13,14]. Recently, brassinosteroids were suggested to promote branching through the direct transcriptional regulation of *BRANCHED1* (*BRC1*) by the BR signalling component BRASSINAZOLE-RESISTANT1 (BZR1) [15].

Over the past two decades, a new group of plant hormones, strigolactones (SLs), has been identified as suppressors of plant branching [9,16]. Recently, great advances have been made in SL signaling and transduction in model plants, and a working model has been established [17,18]. In detail, the receptor DWARF (D14) perceives and hydrolyses SLs and then physically interacts with an F-box protein, MORE AXILLARY GROWTH2 (MAX2), to form an SCF complex [19,20,21]. Subsequently, this SCF complex promotes the ubiquitination and degradation of transcriptional repressors, including SUPPRESSOR OF MAX2-LIKE 6 (SML6), SML7, and SML8 [22,23]. The degradation of these suppressors releases the expression of their downstream genes, such as the extensive plant branching inhibitors BRC1 and TEOSINTE BRANCHED1 (TB1) [24,25,26].

Dissimilar to other hormone receptor genes, *D14* encodes the α/β hydrolase that hydrolyses SLs; subsequently, D14 produces an active D-ring-induced intermediate molecule (CLIM) and initiates SL signaling by irreversibly binding the SLs to CLIM [21]. This new “substrate-enzyme-active molecule-receptor” provides a new direction for plant hormone research. Since the establishment of the D14-MAX2 module, *D14* has been identified in many plants, including wheat [27], apple [28], barley [29], pea [30], and soybean [31]. In alfalfa, studies have revealed a variety of branching regulators, most of which participate in alfalfa branching through the miR156-SPL module [3,32,33]. Nevertheless, branching regulators involved in SL signaling and transduction remain poorly understood in alfalfa.

Here, we reported the characterization of the strigolactone receptor gene *D14* in alfalfa. Quantitative real-time PCR suggested that the expression levels of *MsD14* were much higher in the flowers, roots, and seedpods than those in other tissues, consistent with the GUS staining results. The next studies indicated that *MsD14* could significantly respond to strigolactone in alfalfa seedlings, and its protein was located in the nucleus, cytoplasm, and cytomembrane. Significantly, we confirmed that silencing of *D14* in alfalfa increased biomass by promoting branching through the alfalfa transformation assay. Moreover, the Y2H assay revealed that MsD14 could physically interact with AtMAX2 and MsMAX2. Our findings suggested the presence of a conserved D14-MAX2 module in alfalfa branching regulation and provided candidate genes for alfalfa high-yield molecular breeding.

## 2. Results

### 2.1. Identification of the Strigolactone Receptor Gene (D14) in Alfalfa

Using the sequences of *Arabidopsis AtD14* and rice *OsD14 as* queries, we searched the alfalfa XinJiangDayYe cDNA database (https://figshare.com/articles/dataset/genome_fasta_sequence_and_annotation_files/12327602 4 March 2022). Alignments showed that there were four similar sequences in the cDNA database that differed by several SNPs (Figure S1). We cloned *MsD14* from cDNA and genomic DNA. Sequence alignment demonstrated that *MsD14* harbored two exons and one intron, and the CDS of *MsD14* was 710 bp, encoding 300 amino acid residues.

**Figure 1:**
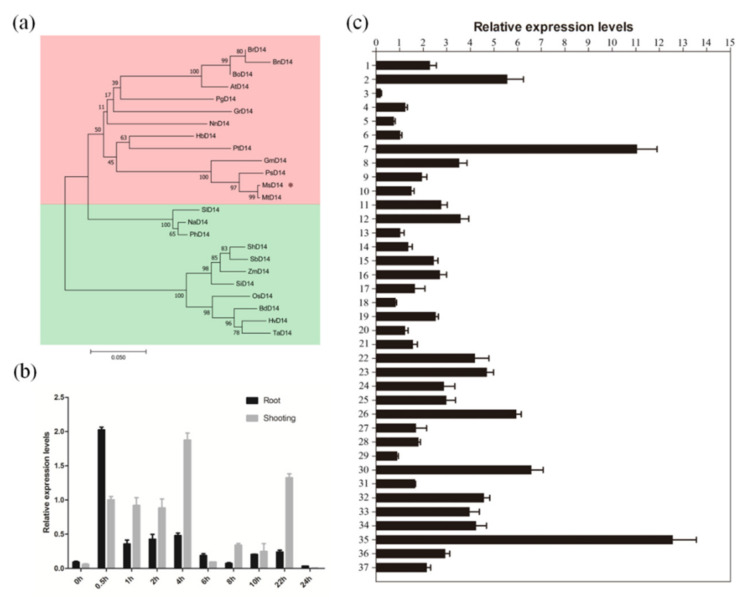
Characterization of *MsD14*. (**a**) Phylogenetic analysis of MsD14 among plants. The accession number of D14 protein used for phylogenetic analysis is listed as follows. BrD14: Brassica rapa (XP_009130408); BnD14: *Brassica napus* (CDY42894); BoD14: *Brassica oleracea* (XP_013638430); AtD14: *Arabidopsis thaliana* (NP_566220); PgD14: *Punica granatum* (OWM70752); GrD14: *Gossypium raimondii* (XP_012451974); NnD14: *Nelumbo nucifera* (XP_010248100); HbD14: *Hevea brasiliensis* (XP_021646820); PtD14: *Populus trichocarpa* (XP_002302409); GmD14: *Glycine max* (XP_003557012); PsD14: *Pisum sativum* (AMB61024); MtD14: *Medicago truncatula* (XP_003589086); SlD14: *Solanum lycopersicum* (XP_004238093); NaD14: *Nicotiana attenuata* (XP_019258478); PhD14: *Petunia hybrida* (AFR68698); ShD14: *Saccharum hybrid* (AJY78078); SbD14: *Sorghum bicolor* (XP_002468316); ZmD14: *Zea mays* (NP_001150635); SiD14: *Setaria italica* (XP_004985292); OsD14: *Oryza sativa* (XP_015631400); BdD14: *Brachypodium distachyon* (XP_003558555); HvD14: *Hordeum vulgare* (AJP07999); TaD14: *Triticum asetivum* (AK332360). * indicates D14 protein in *Medicago Sativa*. (**b**) The relative expression level of *MsD14* in response to GR24. (**c**) Temporal and spatial expression pattern of *MsD14* in alfalfa; 1–2 represent the roots and leaves in seedling stage; 3–6, roots, necks, leaves, and branches in vegetative stage; 7–9 represent roots, stems, and leaves in regreening stage; 10–21 represent heads, nodes, leaves, and stems in the early, middle, and later branching stages; 22–29 represent flowers, nodes, leaves, and stems in the squaring and early flowering stages; 30–34 represent heads, flowers, nodes, leaves, and stems in the flowering stage; 35–37 represent the seedpods at 0, 3, and 5 DAF.

We compared the sequence similarities between MsD14 and D14 in *Arabidopsis* and rice, and the results showed that MsD14 harbored high similarities with AtD14 (95%) and pOsD14 (90%), respectively (Figure S2). The ohylogenetic analysis of *D14* among plants suggested that *MsD14* was the most closely related to *MtD14* in *Medicago truncatula*, consistent with the evolutionary relationship of these species (Figure 1a). Moreover, *D14* genes from dicotyledon and monocotyledon plants were clustered into different groups, indicating that *D14* existed before the divergence of dicots from monocots and then evolved independently (Figure 1a).

In addition, we measured the relative expression levels of *MsD14* in response to strigolactone, as *D14* is a strigolactone receptor gene. The results showed that *MsD14* significantly responded to strigolactone in alfalfa seedlings (Figure 1b). Interestingly, the expression of *MsD14* was dramatically elevated in roots at 0.5 h and then sharply decreased, while the peak transcript levels in shoots occurred at 4 h, suggesting that the response of *MsD14* in roots was more sensitive than that in shoots.

### 2.2. Temporal and Spatial Expression Patterns of MsD14

To clarify the roles of *MsD14* in alfalfa growth and development, we investigated the expression patterns of *MsD14* through qRT-PCR in 37 different alfalfa tissues: roots, stems, leaves, apical meristems, and flowers at different growth stages (seedling, regreening, branching, squaring, and flowering stages), as well as seedpods collected at 0, 3, and 5 days after fertilization (DAF). The expression profile showed that *MsD14* had the highest expression levels in seedpods at 0 DAF and the lowest expression in roots at the seedling stage (Figure 1c). Furthermore, the relative expression levels of *MsD14* in flowers and roots at the regreening stage were much higher than those in the other tissues, demonstrating that this gene might play critical roles in those tissues.

**Figure 2:**
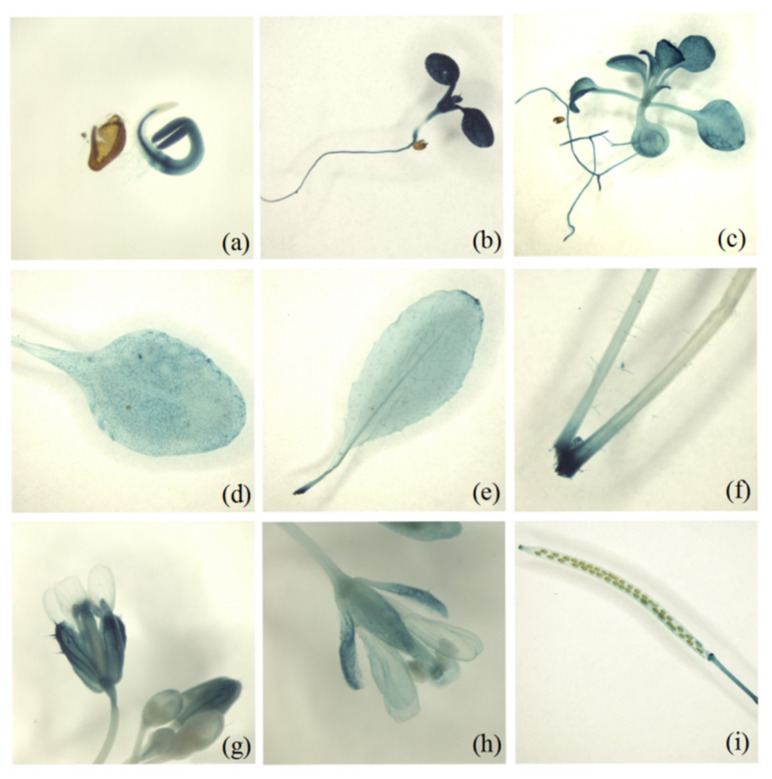
Histochemical localization of GUS activity in pMsD14-GUS transgenic *Arabidopsis* plants. (**a**–**i**) Seed that germinated for 72 h, 7-day, and 15-day-old seedlings; 25- and 35-day-old rosette leaf stem; inflorescence; flower; and seedpod for *OsD14 as*.

In addition, we constructed a chimeric gene consisting of the *MsD14* promoter fused to the GUS (β-glucuronidase) reporter gene, transformed the construct into *Arabidopsis*, and then tested its expression in various tissues. The histochemical analysis of the GUS activity in transgenic *Arabidopsis* showed that *MsD14* was highly expressed in germinating seeds, seedlings, leaves, flowers, and seedpods, while it showed a low expression in stems and petals (Figure 2). These GUS staining results were similar to our qRT-PCR results, indicating that *MsD14* was primarily expressed in roots, flowers, and seedpods.

### 2.3. Subcellular Localization of MsD14

**Figure 3:**
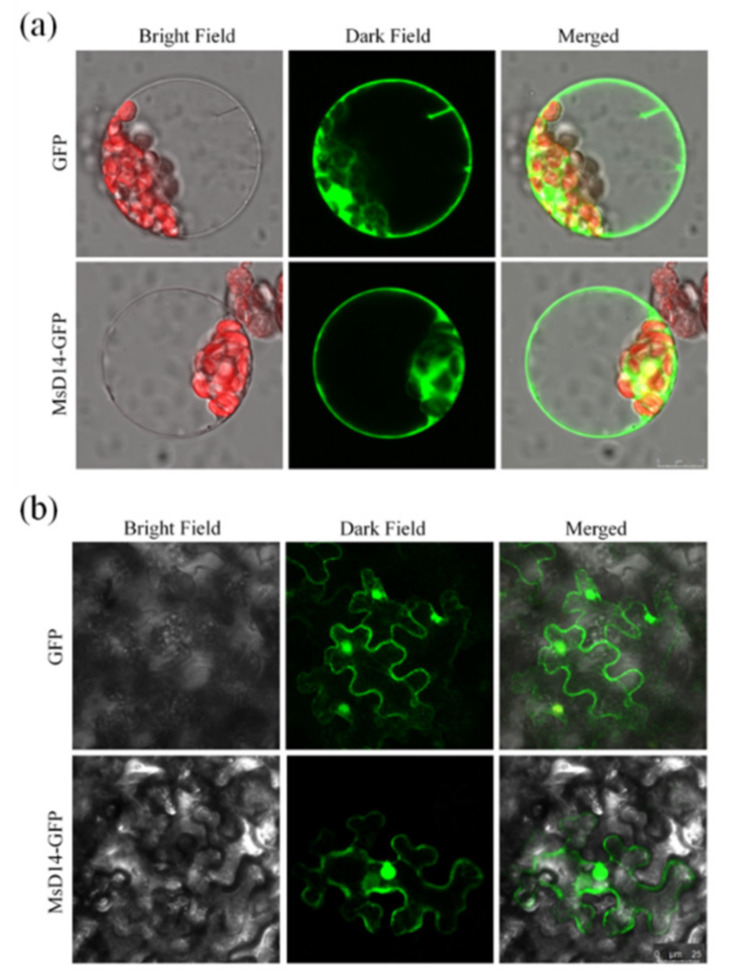
The subcellular localization of MsD14 in *Arabidopsis* protoplast (**a**) and tobacco epidermal cells (**b**). GFP represented the independent GFP protein; MsD14-GFP represented the fusion protein of MsD14 and GFP.

Previous studies have shown that both *AtD14* and *OsD14* were located in the nucleus and cytoplasm; therefore, we evaluated the subcellular localization of *MsD14* in both *Arabidopsis* protoplast and tobacco (*Nicotiana benthamiana*) epidermal cells. To this end, we generated pJIT163::*MsD14-GFP* for the *Arabidopsis* protoplast transformation and pCAMBIA1302::*MsD14-GFP* for tobacco epidermal cells transformation. The results indicated that MsD14-GFP fusion protein fluorescence was targeted exclusively to the nucleus, cytoplasm, and cytomembrane in both *Arabidopsis* protoplast and tobacco epidermal cells, and control GFP fluorescence was observed in the entire cell (Figure 3).

### 2.4. Silencing of MsD14 Increased the Branching Number in Alfalfa

As the *d14* mutant promotes branching in *Arabidopsis* and rice [34,35], we hypothesized that *MsD14* was a key regulator of branching in alfalfa. We obtained transgenic alfalfa that were overexpressed or were silenced for *MsD14* via *Agrobacterium*-mediated transformation. qRT-PCR showed that the relative expression levels of *MsD14* in the overexpression lines were 10–60-fold higher than those in the control plants, while the transcript levels of *MsD14* in the silenced lines decreased to various levels, with a reduction as low as 94% (Figure S3). Phenotypic assessment indicated that only the transgenic plants with dramatically reduced *MsD14* expression (RNAi-2, RNAi-5, and RNAi-7) showed increased branching (Figure S3). However, the branching number of the overexpression lines was not significantly different from that of the control plants (Figure S3).

**Figure 4:**
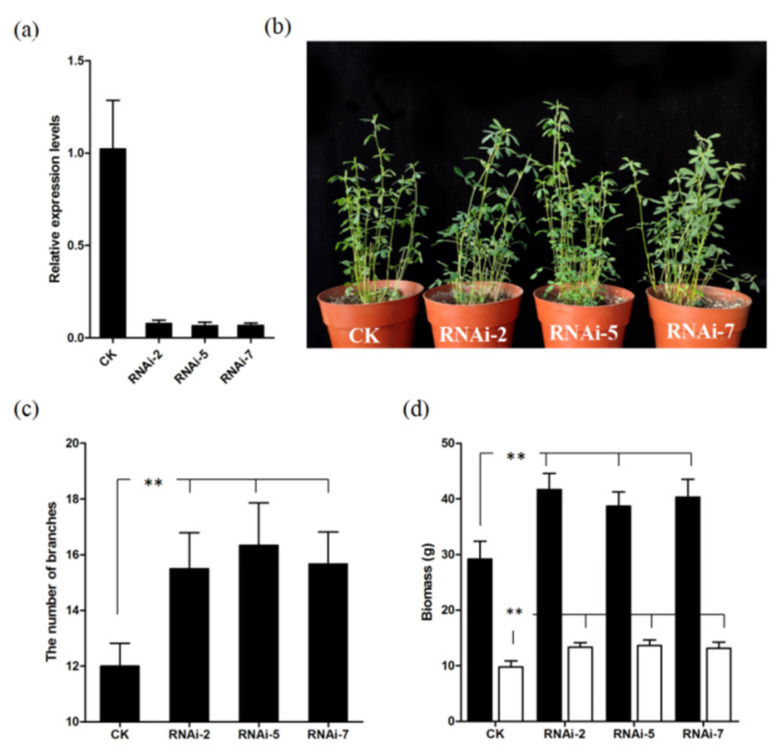
Phenotypic analyses of transgenic plants. (**a**) The relative expression levels of *MsD14*; (**b**) the representative phenotypes of transgenic plants; (**c**) the number of branches of transgenic plants; (**d**) the fresh and dry biomass of transgenic plants. ** indicates a significant difference at *p* < 0.01 level.

For further characterization, three transgenic lines (RNAi-2, RNAi-5, and RNAi-7) with more than 90% reduction of *MsD14* expression levels were selected for detailed phenotypic assessment (Figure 4a). The results showed that the transgenic plants exhibited a 29.16–36.11% increase in branching number, which was significantly different from the results for the control plants (Figure 4b,c). Further studies on biomass indicated that the transgenic plants showed a 32.57–42.85% increase in fresh biomass and a 34.52–39.54% increase in dry biomass compared with the control plants (Figure 4d). These results strongly suggested that silencing *MsD14* positively affected biomass by increasing the branching number in alfalfa.

### 2.5. Interaction of MsD14 with MAX2

Previous studies have shown that D14 could physically interact with MAX2 in the presence of strigolactone in *Arabidopsis* and rice [20,22]. Thus, we speculated that there could be such an interaction between MsD14 and the reported AtMAX2 from *Arabidopsis*, as well as MAX2 from alfalfa. In this context, we identified the *MsMAX2* gene from alfalfa based on the sequences of *AtMAX2* and *OsD3*. The analysis revealed that the MsMAX2 protein candidate shared a high-level identity with AtMAX2 (95%) and OsD3 (91%) (Figure S4). Next, we conducted a yeast two-hybrid assay to investigate the presumed interaction. The results showed that MsD14 could interact with AtMAX2 and MsMAX2 in the presence of GR24, whereas the interactions had not existed when the GR24 was absent (Figure S5). These results confirmed the conservation of the D14-MAX2 regulatory module in both *Arabidopsis* and alfalfa.

## 3. Discussion

In the past two decades, it SLs in plant growth and development have been a popular research topic, especially shoot branching [36,37]. However, the SL receptor D14 was recently characterized as an α/β hydrolase, which is completely different from other plant hormone receptors [21]. In this study, we isolated the homologous *D14* gene in alfalfa. The sequence alignment of *D14* genes showed a high sequence similarity among *Arabidopsis*, rice, and alfalfa, indicating that the obtained *D14* was the SL receptor gene in alfalfa (Figure S2). Further evidence showed that the expression of *MsD14* was significantly responsive to strigolactone in alfalfa seedlings, with a dramatically rapid response in the roots (Figure 1b).

It is well known that *D14* negatively regulates shoot branching in several plants. In *Arabidopsis*, the *d14* mutant showed recessive tillering and dwarf phenotypes, consistent with the phenotype of the rice *dwarf 14* mutant (*d14*) [34,35]. The *dad2* mutant is the *D14* mutant in petunia, which also shows an increased branching phenotype [38]. Based on these results, we speculated that a mutation in *MsD14* would also have an effect on alfalfa branching. To verify the above hypothesis, we conducted *Agrobacterium*-mediated transformation assays. As expected, the transgenic alfalfa plants with silencing of *MsD14* showed an increased branching number (Figure 4). However, transgenic plants overexpressing *MsD14* showed no significant differences in branching number or other traits compared with the control plants (Figure S3).

Branching is the main biomass component in alfalfa. The correlation between branching number and biomass yield was much higher than that between the branching number and other biomass components [39,40]. Silencing of *MsD14* resulted in an increased branching number and enhanced biomass yield, suggesting its critical role in alfalfa biomass formation. As the successful establishment of a gene editing system in alfalfa, editing in *MsD14* showed a huge potential application in alfalfa high biomass yield breeding in the future [41].

**Figure 5:**
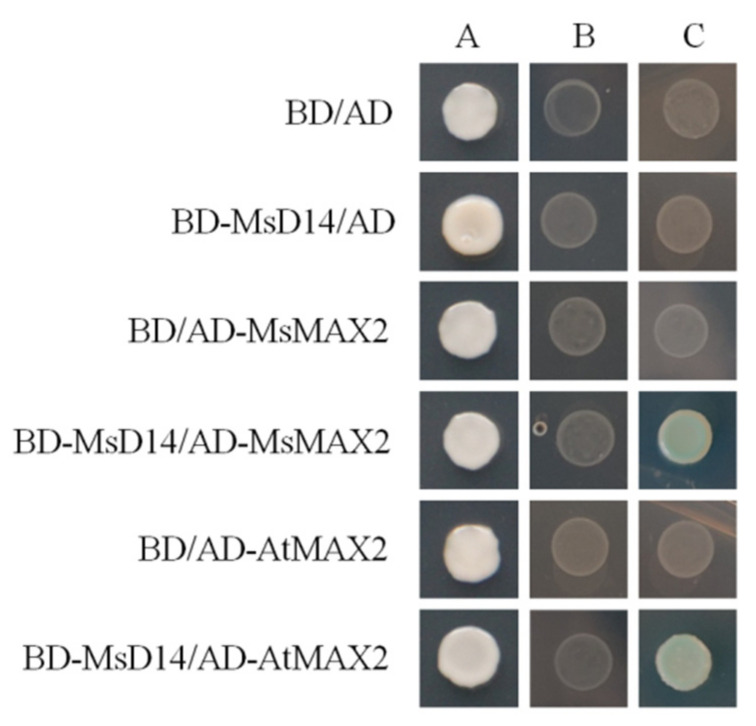
The interaction between MsD14 with MsMAX2 and AtMAX2. (**A**–**C**) SD/-L/-W, SD-L/-W/-H/-A+X-A-gal, and SD-L/-W/-H/-A+X-a-gal+GR24, respectively.

The molecular regulatory mechanism of SLs in branching development has been established in model plants [19,20,26]. The conserved D14-MAX2 module has been identified in other plants [42,43,44]. In our research, we proved that in the absence of SLs, MsD14 could physically interreact with AtMAX2, as well as its homologous protein MsMAX2, in alfalfa (Figure 5). Combined with the results from the transformation assays, we proposed a possible working model of *MsD14* in alfalfa branching regulation. In this working model, we proposed that MsD14 and MsMAX2 act as the core modules and participate in alfalfa branch development with other putative regulatory factors.

Even though homologous genes play similar roles in plants, functional differentiation always exists between different types of plants. A typical case is the *SPL3* homologous gene. *AtSPL13* controls floret development; however, its orthologue in rice, *OsSPL13*, positively regulates grain size by influencing cell proliferation [45,46]. In our study, subcellular localization of MsD14 in both *Arabidopsis* protoplast and tobacco epidermal cells showed that MsD14 was located in the nucleus, cytoplasm, and cytomembrane, which was inconsistent with the subcellular localization of AtD14 and OsD14 (Figure 3). This difference suggested the possible differentiation between MsD14 and other SL receptors. We absolutely confirmed that *MsD14* controls alfalfa branching through the D14-MAX2 module, but the detailed regulatory mechanism should be further studied.

Besides branching development, SLs have been indicated to participate in a series of plant growth and development processes, including seed germination and development [47,48], stem elongation [49], leaf expansion [50], leaf senescence [51], drought and salinity responses [52], and nodule development [53]. In the present study, we also observed that *MsD14* was primarily expressed in the roots, flowers, and seedpods (Figure 1, 2). These results suggested that *MsD14* functions not only in branching development, but also in other stages of alfalfa growth and development, especially in flowering and seed development. The multiple functions of *MsD14* should be further investigated in future studies.

## 4. Materials and Methods

### 4.1. Plant Materials and Growth Conditions

The alfalfa genotype, CV. Zhongmu No. 1, was used for genetic transformation. It was grown in a greenhouse at 25 °C with 16 h of light/day, and transgenic plants were obtained. Other alfalfa CV. Zhongmu No. 1 seeds were surface sterilized with 75% (*v*/*v*) for 5 min, rinsed five times with sterile water, and then placed on moistened filter paper in a dish. Ten-day-old seedlings were then used for the different experiments. For each experiment, the collected samples were immediately frozen in liquid nitrogen and stored at −80 °C for RNA isolation.

The seedlings were transferred into pots (30 cm in diameter) full of nutritional soil, grown in the same greenhouse for two months, and were then moved outdoors to grow. Roots, stems, blades, branches, buds, flowers, and seedpods were collected from three independent plants for temporal and spatial expression analyses of *MsD14*.

For the expression analysis of *MsD14* in response to strigolactones (GR24), seedlings were cultured in 1/2 MS liquid medium and were grown in a controlled growth cabinet under a 16/8 h light/dark regime at 25 °C. The plants were watered with 1 mmol·L^−1^ GR24 after their fourth leaf blade expanded. The roots and shoots were separated and collected at 0, 2, 24, and 72 h.

### 4.2. Nucleic Acid Extraction and PCR

Genomic DNA was extracted from young leaves using the modified CTAB method [54]. PCR was performed in a total volume of 20 μL, and each reaction contained 50 ng of DNA, 1 μL each of 5 mmol L^−1^ forward and reverse primers, and 10 μL of 2 × Taq PCR Mix (Genstar, code: A012). All of the primers used in this study are listed in Table S1.

The total RNA was isolated from various alfalfa tissues with an Eastep^TM^ Super Total RNA Extraction Kit (Promega, code: LS1040), and the cDNA was then synthesized with TransScript One-Step gDNA Removal and cDNA Synthesis SuperMix (TransGen, code: AT311) following the manufacturer’s instructions. Quantitative RT–PCR (qRT–PCR) was conducted with SYBR Green on a 7500 Real-time PCR system (Applied Biosystems, Foster City, CA, USA) in a total volume of 20 μL, and each reaction contained 2 μL of cDNA, 1 μL of 5 mmol L^−1^ gene-specific primers, and 10 μL of 2 × RealStar Green Power Mixture (Genstar, code: A311).

### 4.3. MsD14 Cloning and Informatics Analysis

Full-length MsD14 was PCR-amplified from alfalfa cDNA and genomic DNA using the primers MsD14-CDS. The gene structure was determined by aligning the genomic DNA and its corresponding CDS. D14 gene sequences from other plants were downloaded from NCBI, and the sequence alignment and phylogenetic analysis were conducted using MEGA 7.0 software [55].

### 4.4. GUS Histochemical Assay

The 2-kb MsD14 promoter was PCR-amplified from alfalfa genomic DNA with the primer pairs MsD14-PRO and was sub-cloned into the pCAMBIA1391z vector at its *Sal*I and *EcoR*I sites to generate the pCAMBIA1391z::p*MsD14-GUS* recombinant construct. The verified construct was mobilized into *Agrobacterium* strain GV3101 and transferred into wild-type (Col-0) *Arabidopsis* through the floral dip method [56]. Mature homozygous T_3_ seeds were used for histochemical localization of the GUS activity, as previously described [57].

### 4.5. Subcellular Localization Analysis

The full-length MsD14 CDS without a stop codon was cloned using the primers MsD14-GFP. The resulting fragment was introduced into a pJIT163 and pCAMBIA1302 vector, which lined by *Hind*III and *BamH*I to generate the pJIT163::*MsD14-GFP* and pCAMBIA1302::*MsD14-GFP* recombinant construct, respectively. For *Arabidopsis* protoplast transformation, the verified plasmid and the control were each introduced into an *Arabidopsis* protoplast using PEG4000, as previously described [54]. For tobacco (*Nicotiana benthamiana*) epidermal cell transformation, the verified plasmid and control were co-infiltrated into tobacco leaves and analyzed 48 h after infiltration. Subcellular localization of GFP was then monitored using a laser scanning confocal microscope (Leika TCS-NT, Wetzlar, Germany).

### 4.6. Alfalfa Expression Vector Construction and Genetic Transformation

The full-length MsD14 CDS was sub-cloned into pbi121ΔGUS at its XbaI and SmaI to generate the overexpression construct pbi121ΔGUS::*MsD14*. For RNAi vector construction, a 400-bp fragment of MsD14 was PCR-amplified from the MsD14 CDS using the primer pairs MsD14-RNAi. The resulting fragment was then inserted into pDONER221 and transferred into pK7GWIWG2D(II) by attL/attB recombination to generate RNAi::*MsD14*. The verified recombinant constructs were separately transferred into *Agrobacterium* strain GV3101 using the freezing/heat-shock method. *Agrobacterium*-mediated transformation was used to obtain transgenic alfalfa plants, as previously reported [58].

### 4.7. Yeast Two-Hybrid Assays

Yeast two-hybrid assays were used to check the interaction of MsD14 and MAX2. The CDS of MsD14 was sub-cloned into pGBKT7 at its *Nde*I and *EcoR*I sites to generate pGBKT7::MsD14, which is designated as BD-MsDA14. The CDS of AtMAX2 and MsMAX2 were PCR-amplified using the primers AtMAX2-CDS and MsMAX2-CDS, and sub-cloned into pGADT7 at its *Nde*I and *EcoR*I sites generate AD-AtMAX2 and AD-MsMAX2. Both bait (BD-MsDA14) and prey (AD-AtMAX2 and AD-MsMAX2) were co-transferred into the Y2H Gold yeast strain and were selected on a synthetic dextrose medium lacking Leu and Trp (SD -L/W). The selected yeast cells were transferred to the SD medium lacking Leu, Trp, His, and Ade (SD -L/W/H/A) for the interaction analysis.

## 5. Conclusions

In summary, we firstly characterized the strigolactone receptor gene *MsD14* and investigate its bio-function in alfalfa. Silencing of *MsD14* in alfalfa increased the biomass by promoting branching through the conserved D14-MAX2 module, suggesting the critical role of *MsD14* in alfalfa branching regulation. Our findings provide candidate genes for alfalfa high-yield molecular breeding.

## Data Availability

Data are contained within the article or Supplementary Materials.

## Supplementary Materials

The following are available online at https://www.mdpi.com/article/10.3390/plants11070939/s1. Figure S1: Sequence alignment of four *MsD14* cDNA. Figure S2: Amino acid sequence analysis of MsD14 with AtD14 and OsD14. Figure S3: Phenotypic analyses of transgenic alfalfa plants. The relative expression levels of *MsD14* in overexpression lines (a) and down-regulation lines (b). (c) The representative phenotypes of transgenic alfalfa plants. (d) The number of branches of transgenic alfalfa plants. Figure S4: Amino acid sequence analysis of MsMAX2 with AtMAX2 and OsD3. Table S1: Primers used in this study.
