# Supplementary material for: Silencing of MsD14 Resulted in Enhanced Forage Biomass through Increasing Shoot Branching in Alfalfa (Medicago sativa L.)
Source: Plants (Basel). 2022 Mar 30;11(7):939. doi: 10.3390/plants11070939 (PMC9003486; doi:10.3390/plants11070939)
Supplement: Supplementary file 1 [file plants-11-00939-s001.zip › Supplementary Figures.pdf]

```
MsD14-1  ATGGGCAGCACTTCCATCCTCGACGCACTCAATGTCCGCGTAGAAGGCTCCGGCGACAAATACATCGTCTTCGCTCACGG  80
MsD14-2  ATGGGCAGCACTTCCATCCTCGACGCACTCAATGTCCGCGTAGAAGGCTCCGGCGACAAATACATCGTCTTCGCTCACGG  80
MsD14-3  ATGGGCAGCACTTCCATCCTCGACGCACTCAATGTCCGCGTAGAAGGCTCCGGCGACAAATACATCGTCTTCGCTCACGG  80
MsD14-4  ATGGGCAGCACTTCCATCCTCGACGCGCTCAATGTCCGCGTAGAAGGCTCCGGCGACAAATACATCGTCTTCGCTCACGG  80

MsD14-1  CTTCGGCACCGACCAATCAGCATGGCAGCGCGTGCTCCCTTACTTCACCCGCAGCTACAAAGTCATTCTCTATGACCCCG  160
MsD14-2  CTTCGGCACCGACCAATCAGCATGGCAGCGCGTGCTCCCTTACTTCACCCGCAGCTACAAAGTCATTCTCTATGACCTCG  160
MsD14-3  CTTCGGCACCGACCAATCAGCATGGCAGCGCGTGCTCCCTTACTTCACCCGCAGCTACAAAGTCATTCTCTATGACCTCG  160
MsD14-4  CTTCGGCACCGACCAATCAGCATGGCAGCGCGTGCTCCCTTACTTCACCCGCAGCTACAAAGTCATTCTCTATGACCTCG  160

MsD14-1  TTTGCGCCGGCAGTGTCAACCCCGATTACTTTGATTACCGCCGTTACACAACTCTTGACGCTTACGTTGATGATCTCCTC  240
MsD14-2  TTTGCGCCGGCAGTGTCAACCCCGATTACTTTGATTACCGCCGTTACACAACTCTTGACGCTTACGTTGATGATCTCCTC  240
MsD14-3  TTTGTGCCGGCAGTGTCAACCCCGATTACTTTGATTACCGCCGTTACACAACTCTTGACGCTTACGTTGATGACCTCCTC  240
MsD14-4  TTTGCGCCGGCAGTGTCAACCCCGATTACTTTGATTACCGCCGTTACACAACTCTTGACGCTTACGTTGATGACCTCCTC  240

MsD14-1  AACATCCTTGATTCCCTCCACGTCACTCGCTGTGCTTACGTCGGTCACTCCATCTCCGCCATGATCGGAATGCTAGCTTC  320
MsD14-2  AACATCCTTGATTCCCTCCATGTCACTCGCTGTGCTTACGTCGGTCACTCCATCTCTGCCATGATCGGAATGCTAGCTTC  320
MsD14-3  AACATCCTTGATTCCCTCCATGTCACTCGCTGTGCTTACGTCGGTCACTCCATCTCCGCCATGATCGGAATGCTAGCTTC  320
MsD14-4  AACATCCTTGATTCCCTCCATGTCACTCGCTGTGCTTACGTCGGTCACTCCATCTCCGCCATGATCGGAATGCTAGCTTC  320

MsD14-1  CATTCGCCGCCCTGAGCTCTTCTCCAAACTCATCCTTATCGGTGCCTCCCCAAGATTTTTGAACGACGGTGAAAATTACC  400
MsD14-2  CATTCGCCGCCCTGAGCTCTTCTCCAAACTCATCCTTATCGGTGCCTCCCCAAGATTTTTGAACGACGGTGAAAATTACC  400
MsD14-3  CATTCGCCGCCCTGAGCTCTTCTCCAAACTCATCCTTATCGGTGCCTCCCCAAGATTTTTGAACGACGGTGAAAATTACC  400
MsD14-4  CATTCGCCGCCCTGAGCTCTTCTCCAAACTCATCCTTATCGGTGCCTCCCCAAGATTTTTGAACGACGGTGAAAATTACC  400

MsD14-1  ACGGAGGATTCGAGCAGGGAGAAATTGAGCAAGTTTTTCAGCAATGGAAGCAAACTATGAAGCGTGGGTGAACGGTTTT  480
MsD14-2  ACGGAGGATTCGAGCAGGGAGAAATTGAGCAAGTTTTTCAGCAATGGAAGCAAACTATGAAGCGTGGGTGAACGGTTTT  480
MsD14-3  ACGGAGGATTCGAGCAGGGAGAAATTGAGCAAGTTTTTCAGCAATGGAAGCAAACTATGAAGCGTGGGTGAACGGTTTT  480
MsD14-4  ACGGAGGATTCGAGCAGGGAGAAATTGAGCAAGTTTTTCAGCAATGGAAGCAAACTATGAAGCGTGGGTGAACGGTTTT  480

MsD14-1  GCTCCACTGGCTGTCGGGGCCGATGTTCCGACAGCTGTTCGAGAATTTTCTAGAACGCTCTTTAACATGAGACCAGATAT  560
MsD14-2  GCTCCACTGGCTGTCGGGGCCGATGTTCCGACAGCTGTTCGAGAATTTTCTAGAACGCTCTTTAACATGAGACCAGATAT  560
MsD14-3  GCTCCACTGGCTGTCGGGGCCGATGTTCCGACAGCTGTTCGAGAATTTTCTAGAACGCTCTTTAACATGAGACCAGATAT  560
MsD14-4  GCTCCACTGGCTGTCGGGGCCGATGTTCCGACAGCTGTTCGAGAATTTTCTAGAACGCTCTTTAACATGAGACCAGATAT  560

MsD14-1  ATCTCTCTTTGTTTCAAGGACAGTTTTCAACAGTGATCTGAGAGGGATTCTAGGACTGGTTAAGGTACCTTGTTGTATCA  640
MsD14-2  ATCTCTCTTTGTTTCAAGGACAGTTTTCAACAGTGATCTGAGAGGGATTCTAGGACTGGTTAAGGTACCTTGTTGTATCA  640
MsD14-3  ATCTCTCTTTGTTTCAAGGACAGTTTTCAACAGTGATCTGAGAGGGATTCTAGGACTGGTTAAGGTACCTTGTTGTATCA  640
MsD14-4  ATCTCTCTTTGTTTCAAGGACAGTTTTCAACAGTGATCTGAGAGGGATTCTAGGACTGGTTAGGGTACCTTGTTGTATCA  640

MsD14-1  TGCAAACGGCGAGAGACATGTCGGTTCCGGCGACGGTGGCGACGTACATGAAGGAGCATCTTGGCGGGAAGAGTACGGTG  720
MsD14-2  TGCAAACGGCGAGAGACATGTCGGTTCCGGCGACGGTGGCGACGTACATGAAGGAGCATCTTGGCGGGAAGAGTACGGTG  720
MsD14-3  TGCAAACGGCGAGAGACATGTCGGTTCCGGCGACGGTGGCGACGTACATGAAGGAGCATCTTGGCGGGAAGAGTACGGTG  720
MsD14-4  TGCAAACGGCGAGAGACATGTCGGTTCCGGCGACGGTGGCGACGTACATGAAGGAGCATCTTGGCGGGAAGAGTACGGTG  720

MsD14-1  CAGTGGCTGGACACGGAGGGGCATCTTCCTCATTTGAGTGCTCCTTCTTACTTGGCTCATCAACTGGAGATAGCACTTTC  800
MsD14-2  CAGTGGCTGGACACGGAGGGGCATCTTCCTCATTTGAGTGCTCCTTCTTACTTGGCTCATCAACTGGAGATAGCACTTTC  800
MsD14-3  CAGTGGCTGGACACGGAGGGGCATCTTCCTCATTTGAGTGCTCCTTCTTACTTGGCTCATCAACTGGAGATAGCACTTTC  800
MsD14-4  CAGTGGCTGGACACGGAGGGGCATCTTCCTCATTTGAGTGCTCCTTCTTATTTGGCTCATCAACTGGAGATAGCACTTTC  800

MsD14-1  ACAGTAG  807
MsD14-2  ACAGTAG  807
MsD14-3  ACAGTAG  807
MsD14-4  ACAGTAG  807
```

Figure S1: Sequence alignment of the obtained four *MsD14* cDNA.

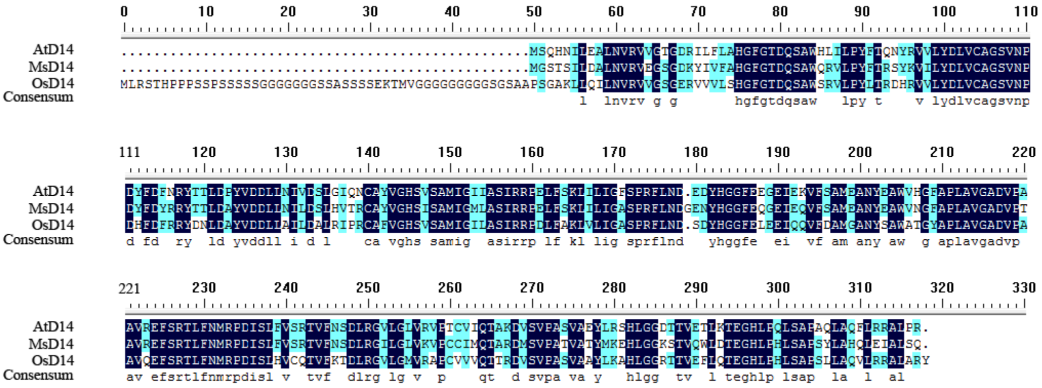

Figure S2: Amino acid sequence alignment of MsD14 with AtD14 and OsD14.

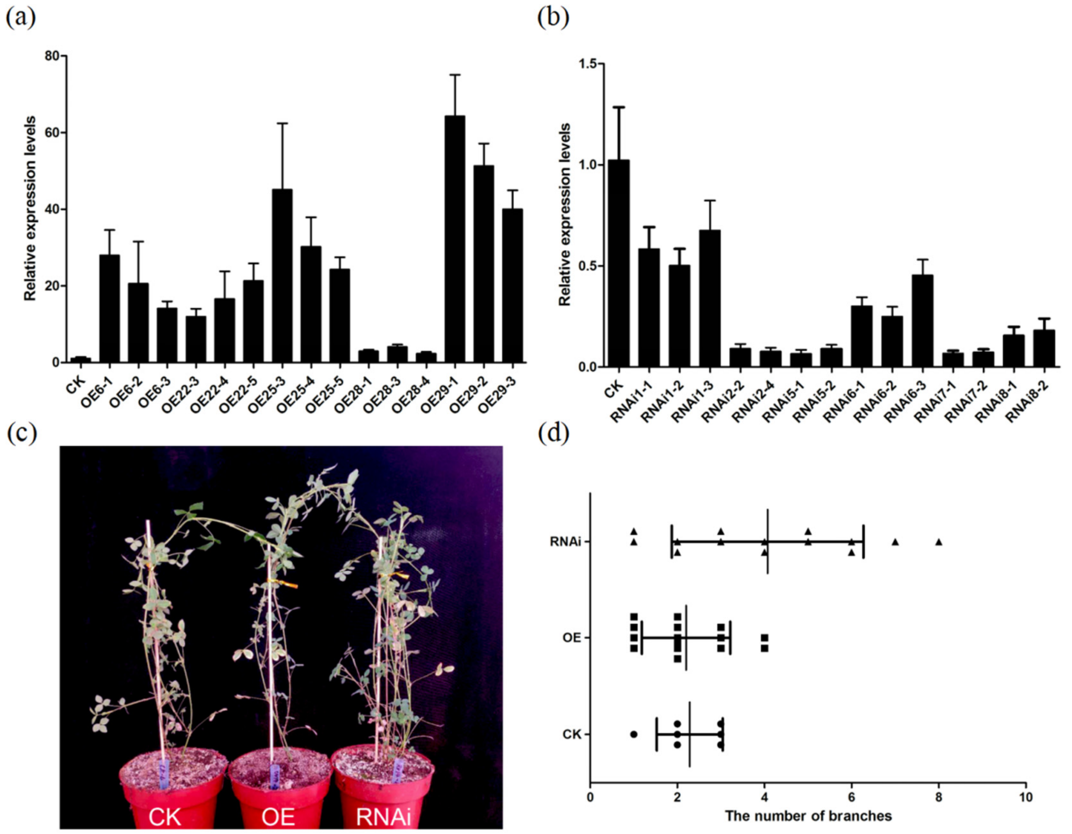

Figure S3: Phenotypic analyses of transgenic plants. The relative expression levels of *MsD14* in overexpression lines (a) and down-regulation lines (b). (c) The representative phenotypes of transgenic plants. (d) The number of branches of transgenic plants.

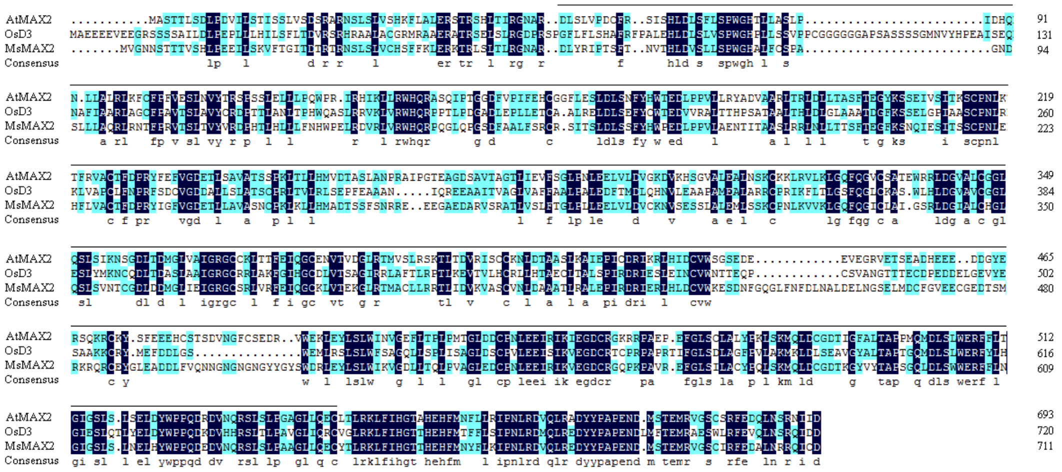

Figure S4: Amino acid sequence alignment of MsMAX2 with AtMAX2 and OsD3.

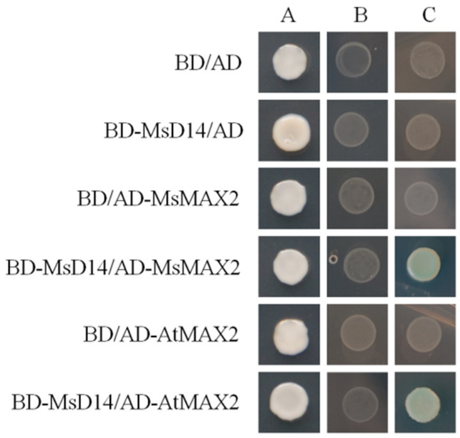

Figure S5: The interaction between MsD14 with MsMAX2 and AtMAX2. A-C represents SD/-L/-W, SD -L/-W/-H/-A+X-A-gal and SD -L/-W/-H/-A+X-a-gal+GR24, respectively.
